# Pericardial Closure Preserves Early Right Ventricular Function After Cardiac Surgery: A Retrospective Cohort Study

**DOI:** 10.3390/jcdd12110431

**Published:** 2025-10-31

**Authors:** Hannah Breuer, Marjolijn C. Sales, Natasja W. M. Ramnath, Yusuf Shieba, Alish Kolashov, Ajay Moza, Lachmandath Tewarie, Rashad Zayat, Nima Hatam

**Affiliations:** 1 Department of Cardiac Surgery, RWTH University Hospital, 52074 Aachen, Germanynramnath@ukaachen.de (N.W.M.R.);; 2 Faculty of Medicine, RWTH University, 52074 Aachen, Germany; 3 Department of Cardiothoracic Surgery, Faculty of Medicine, South Valley University, Qena 83523, Egypt; 4 Department of Cardiothoracic Surgery, Heart Centre Trier, Barmherzigen Brueder Hospital, 54292 Trier, Germany

**Keywords:** pericardial closure, cardiac surgery, right ventricular function, speckle-tracking echocardiography

## Abstract

Background: Perioperative right ventricular (RV) dysfunction is a frequent complication of cardiac surgery linked to poor outcomes and may result from the loss of pericardial support. We investigated whether pericardial closure preserves early postoperative RV function. Methods: We compared patients with pericardial closure versus open pericardium. Co-primary endpoints were early postoperative RV longitudinal function by tricuspid annular plane systolic excursion (TAPSE) and tricuspid annular systolic velocity (TASV). Adjusted comparisons used analysis-of-covariance (postoperative value adjusted for baseline) with prespecified covariates (baseline outcome, LV global longitudinal strain, left-ventricular ejection fraction, LVEDVI, sex, procedure; cardiopulmonary bypass and cross-clamp times when available). Holm correction-controlled multiplicity across the co-primary endpoints. Sensitivity linear mixed-effects models (time × group) were performed. Results: Pericardial closure was associated with better early RV longitudinal function after multivariable adjustment. TAPSE: adjusted mean difference (AMD, Closed–Open) 1.531 mm (95% CI 0.130–2.931; *p* = 0.033). TASV: AMD 1.694 cm/s (95% CI 0.437–2.951; *p* = 0.009; Holm-adjusted *p* = 0.018). Sensitivity analyses yielded consistent estimates. Conclusions: Pericardial closure was independently associated with improved early RV longitudinal function. These adjusted findings address baseline LV imbalances and support considering closure to preserve RV performance; confirmation in prospective trials is warranted.

## 1. Introduction

The right ventricle (RV) is pivotal in exercise tolerance and significantly impacts outcomes following cardiac surgery [1,2]. The function of the right ventricle influences prognosis in patients undergoing cardiac surgery, and the onset of right ventricular failure postoperatively is related to significantly increased morbidity and mortality rates [3].

Perioperative impairment of RV function has important clinical consequences: it is linked to greater requirements for inotropic support, prolonged intensive care unit stays, higher rates of hospital readmission, and an increased risk of in-hospital death and postoperative circulatory collapse [2,4].

Multiple studies report a decline in common two-dimensional echocardiographic indices of RV systolic performance during and immediately after cardiac procedures [4,5]. Longitudinal assessments of RV systolic function, including tricuspid annular plane systolic excursion (TAPSE) and tricuspid annular systolic velocity (TASV/S′), are often reduced after elective coronary artery bypass grafting (CABG), irrespective of the use of cardiopulmonary bypass [6,7]. Proposed explanations for these perioperative changes include loss of pericardial restraint after pericardiotomy, ischemic injury due to suboptimal myocardial protection, and postoperative adhesions or scarring that alter RV geometry and contraction patterns [4,8,9].

Intraoperative transesophageal echocardiography (TEE) investigations have documented reductions in TASV that occur immediately after the pericardium is opened, consistent with anticipated geometric alterations of the RV [10,11]. The conventional mid-esophageal four-chamber TEE view does not consistently allow optimal Doppler alignment with lateral tricuspid annular motion, potentially leading to measurement errors for Doppler-derived velocities [12].

Speckle-tracking echocardiography (STE) employs dedicated software to follow natural acoustic markers in B-mode images and thereby quantifies myocardial displacement, velocity, and strain [13,14]. Unlike tissue Doppler imaging, STE measurements are largely independent of the insonation angle for in-plane motion, avoiding bias introduced by misalignment between the ultrasound beam and the direction of annular or free-wall motion [14]. Consequently, STE may yield more reliable estimates of annular excursion, systolic velocity, and regional deformation when applied perioperatively.

Accordingly, this study used complementary approaches—standard transthoracic echocardiography, speckle-tracking deformation analysis, and color tissue Doppler imaging—to characterize RV systolic performance with greater precision and to determine whether pericardial opening is a principal contributor to the early postoperative decline in longitudinal RV function. We hypothesized that loss of pericardial support produced by surgical opening of the pericardium is a major factor driving reductions in RV longitudinal function during the perioperative and early postoperative period.

## 2. Materials and Methods

### 2.1. Study Design & Patient Population

This cohort study involved a retrospective analysis of data from all patients who underwent cardiac surgery in our department from 2016 to 2020. 1661 patients were screened.

Exclusion criteria were right ventricular dysfunction, RV infarction, RV dilatation (RV end-diastolic diameter ≥ 40 mm), pacemaker stimulation, severe left heart insufficiency (LV-EF ≤ 40%), tricuspid valve insufficiency ≥ 2°, pulmonary valve insufficiency ≥ 2°, atrial fibrillation/flutter, pulmonary hypertension, state after pulmonary artery embolism, chronic obstructive pulmonary disease > GOLD 2, prior cardiac surgery, no transthoracic echocardiography (TTE) pre- and postoperative, insufficient image quality of TTE, and ventricular extrasystoles > 5/min. The patients received coronary artery bypass surgery (CABG), aortic valve replacement (AVR), or a combined procedure (CABG + AVR).

We were able to include 97 patients in total. According to the surgeon’s practice, the patients were then divided into two groups: pericardium left open (Open, *n* = 32) versus pericardium closed at the end of the operation (Closed, *n* = 65).

### 2.2. Data Collection

Preoperatively, a TTE examination was performed to obtain baseline values. Moreover, detailed demographic data, laboratory results, and vital parameters were collected. Cardiac surgery was performed with or without pericardial closure, depending on the surgeon’s preferences. Postoperative outcomes, laboratory, and vital parameters were redetermined. TTE examination was repeated within seven days postoperatively.

### 2.3. Surgical Technique

All operations took place under general anesthesia. Propofol, sufentanil, and a suitable muscle relaxant were used for induction of anesthesia. This was followed by endotracheal intubation and mechanical ventilation (tidal volumes 6–8 mL/kg). Anaesthesia was maintained using appropriate intravenous or inhalative hypnotics and opioids, according to the anaesthesiologist’s preference. In addition, substances for circulatory support (e.g., catecholamines, atropine, and acrinor) were administered if indicated. Blood pressure, electrocardiography (ECG), pulse oximetry, and skeletal muscle tone were monitored by anesthesiology throughout the procedure.

Surgical access was achieved via a standard median sternotomy, then the cardiopulmonary bypass (CPB) was started following direct cannulation of the ascending aorta and right atrium; the surgery was performed under mild hypothermia (32 to 34 °C). Cardiac arrest was induced following aortic clamping with the delivery of cold intermittent blood cardioplegia in an antegrade technique, either via the aortic root or through direct cannulation of the coronary ostia in cases of substantial valve regurgitation. Cardiac arrest was maintained with intermittent, selective delivery of cardioplegia, either antegrade or retrograde, according to the surgeon’s preference.

In the Closed group (*n* = 65), pericardial closure was performed using a continuous suture. When primary tension-free approximation was not feasible, as judged by the operating surgeon, a bovine pericardial patch was used to augment the closure and prevent any cardiac compression.

### 2.4. Echocardiography

Detailed transthoracic echocardiograms (TTEs) were performed by two board-certified physicians using commercially accessible ultrasound machines (GE Vivid E90, GE Vingmed Ultrasound, Horten, Norway) prior to surgery and within seven days postoperatively, with patients positioned in the left lateral decubitus position. In line with current guidelines [15], baseline two-dimensional color pulsed-wave and continuous-wave Doppler echocardiographic parameters were obtained.

TTE images were digitally archived in cine-loop format for subsequent offline processing utilizing Echopac software (EchoPac 204, GE Vingmed Ultrasound, Horten, Norway).

In a cohort of 20 randomly selected patients, the intra- and interobserver variability in the evaluation of cardiac work indices and LV GLS was examined. Sonographers exhibited intra-observer reproducibility with a minimum interval of 5 weeks. Two board-certified sonographers examined inter-observer repeatability employing an identical methodology on double-blind clinical data.

LV dimensions were measured in the parasternal long axis (PLAX). The LV-EF was assessed using the modified biplane Simpson’s method. The left ventricular stroke volume index (LVSVI) was calculated based on the diameter of the LV outflow tract (LVOTd), measured mid-systolic in the PLAX and the velocity-time integral of the LV outflow tract (VTI LVOT), measured using PW Doppler in the apical long axis (APLAX) or in the apical 5-chamber view (5AC). The left ventricular global longitudinal strain (LV GLS) was measured after the endocardial border was scanned using semi-automated speckle-tracking echocardiography from the apical two-, three-, and four-chamber views [16].

RV dimensions were measured in the apical four chamber view (4AC). TAPSE was analysed using M-mode in the 4AC. PW-Tissue Doppler Imaging (PW-TDI) of the lateral tricuspid annulus in the 4AC was used to determine the tissue velocities TASV, respectively, S′, E′ and A′. RV strain and RV strain rate were determined using 2D-STE in 4AC. Only the RV free wall, divided into a basal, a mid-ventricular and a basal segment by the software, was included. Tei index (resp. Myocardial Performance Index, MPI) was calculated using the isovolumetric contraction time (IVCT), ejection time (ET), and isovolumetric relaxation time (IVRT) measured using Tissue Doppler Imaging (TDI) of the lateral tricuspid annulus. In addition, Tei index was determined by measuring the RV ejection time (RV ET) and transpulmonary time using spectral Doppler over the tricuspid valve in 4AC. The transtricuspid flow velocities of the E- and A-waves, as well as the E-deceleration time, were determined end-expiratory by PW Doppler over the tricuspid valve in the 4AC [17]. A subcostal view was chosen to determine the initial V. cava inf. (VCI) diameter approximately 5 mm before the inflow into the right atrium (RA). Further, the right atrial pressure (RAP) was estimated based on the VCI diameter and inspiratory collapse. The presence of the D sign, i.e., diastolic protrusion of the interventricular septum towards the LV, was visually verified in the parasternal short axis (PSAX). To determine pulmonary vascular resistance (PVR), the maximum flow velocity of systolic tricuspid regurgitation (TRVmax) was measured using CW Doppler over the tricuspid valve in 4AC and the velocity-time integral of the right ventricular outflow tract (VTI RVOT) was measured using PW Doppler over the RVOT in PLAX.

### 2.5. Endpoints

The primary endpoint of the study was the preservation of postoperative RV longitudinal function, assessed by the change in tricuspid annular plane systolic excursion (TAPSE) and tricuspid annular systolic velocity (TASV) from baseline to the postoperative measurement. RV function is measured by STE-based RV strain and strain rate. However, other parameters such as RV-FAC, MPI, breath-dependent VCI collapse, hepatovenous flow pattern and trans-tricuspid flow velocities, etc., are used to quantify RVF.

Secondary endpoints of the study are the postoperative LV function after pericardial closure vs. open pericardium, measured by left ventricular echocardiographic parameters such as LV dimensions, SVI, LVEF, and LV GLS, as well as laboratory and vital parameters such as heart rate, hemoglobin, and hematocrit. We also looked at the incidence of postoperative atrial fibrillation comparing the open and closed group.

### 2.6. Ethics

The independent ethics committee of the Medical Faculty at RWTH Aachen University approved the study’s conduct (EK 151/09). The study’s retrospective design precluded the necessity of informed consent as determined by our ethics committee. The criteria of the Declaration of Helsinki of 2004 were observed.

### 2.7. Statistical Analysis

Continuous data are represented as mean ± *SD*. Absolute and relative frequencies are utilized to summarize categorical data. The *t*-test or Wilcoxon-Mann-Whitney test was applied to compare patient groups for continuous data, while Fisher’s exact test or the chi-square test was implemented for categorical variables. To compare the changes in echocardiographic measurements pre- and postoperatively within the groups and between the groups, we used a repeated measurements ANOVA, and we used a Holm-adjusted *p*-value. We conducted Spearman’s correlation to examine the relationship between pericardial closure and the occurrence of postoperative atrial fibrillation. The co-primary endpoints were the early postoperative changes in right-ventricular longitudinal function (TAPSE and TASV). We fitted analysis-of-covariance (ANCOVA) models with group (Closed vs. Open) as the exposure and the following pre-specified covariates to address baseline imbalances: baseline value of the outcome, LV global longitudinal strain (LV GLS), LVEF, LVEDVI, sex, procedure type, and (when available) CPB time and aortic cross-clamp time. We report adjusted mean differences (AMD; Closed–Open) with 95% CIs. As a sensitivity analysis, we used linear mixed-effects models including pre- and postoperative measurements with fixed effects for time, group, and their interaction (time × group) and the same covariates, and a patient-level random intercept. Co-primary endpoints were multiplicity-controlled using Holm; other outcomes were considered exploratory. The significance threshold was set at *p* < 0.05, with all *p* values evaluated as two-tailed. Analyses were performed with STATA (StataCorp, 2019, Stata Statistical Software: Release 16) and the jamovi project (2020, jamovi software, version 2.6.3; JAMovi.org).

## 3. Results

The research was conducted on a total of 97 patients, with 32 patients from the open pericardium group and 65 patients from the closed pericardium group participating in the study. The statistical analysis compared echocardiographic parameters between and within these groups over two time points: preoperatively and postoperatively. The significant findings are summarized in Table 1 and Table 2.

### 3.1. Baseline (Preoperative) Characteristics

At baseline, the open and closed pericardium groups showed several significant differences in left ventricular function. The open pericardium group had a significantly lower (more negative) global longitudinal strain (LV GLS) at −19.2 ± 3.6% compared to −17.4 ± 3.7% in the closed pericardium group (*p* < 0.001). The open group also had a significantly lower left ventricular end-diastolic volume index (LVEDVI) (48.6 ± 11.2 mL/m^2^ vs. 55.7 ± 24.3 mL/m^2^, *p* = 0.007) and a higher left ventricular ejection fraction (LVEF) (58.1 ± 8.4% vs. 55.5 ± 8.9%, *p* = 0.018).

### 3.2. Changes Within Each Group

Both the open and closed pericardium groups showed significant changes in various echocardiographic parameters from the preoperative to the postoperative period, indicating a general decrease in cardiac function following surgery (Table 2).

Open Pericardium Group: Significant reductions were observed in key indicators of RV function, including TAPSE (21.6 ± 3.3 mm pre-op to 12.9 ± 3.1 mm post-op, *p* < 0.001) and tricuspid annular systolic velocity (TASV) (11.7 ± 2.8 cm/s pre-op to 8.3 ± 2.3 cm/s post-op, *p* < 0.001). Left ventricular function also declined, with LV GLS decreasing significantly from −19.2 ± 3.6% to −16.9 ± 3.9% (*p* = 0.006).

Closed Pericardium Group: This group also experienced significant declines in right ventricular function, with TAPSE falling from 22.2 ± 4.4 mm pre-op to 14.6 ± 3.1 mm post-op (*p* < 0.001) and TASV from 12.0 ± 3.5 cm/s to 9.9 ± 2.7 cm/s (*p* < 0.001). Similar to the open group, LV GLS also significantly decreased from −17.6 ± 3.6% to −15.0 ± 3.6% (*p* < 0.001). The closed group also had a significant reduction in stroke volume index (SVI) (37.0 ± 11.5 mL/m^2^ to 31.3 ± 12.0 mL/m^2^, *p* = 0.019) and RV outflow tract velocity time integral (VTI RVOT) (17.9 ± 3.6 cm to 14.6 ± 4.8 cm, *p* < 0.001).

### 3.3. Postoperative Inter-Group Comparison

**Figure 1:**
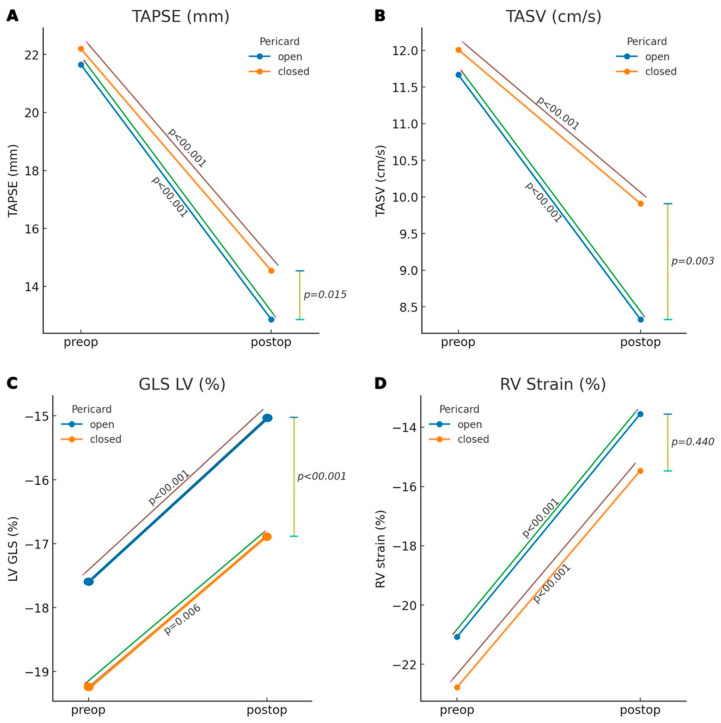
Comparison of the echocardiographic parameters TAPSE, TASV, GLS LV and RV Strain within and between the two groups pre- and postoperatively. (**A**) TAPSE, tricuspid annular plane systolic excursion; (**B**) TASV, tricuspid annular systolic velocity; (**C**) GLS LV, Global longitudinal strain left ventricle; (**D**) RV strain, right ventricular strain.

In the postoperative period, significant differences persisted between the two groups. The open pericardium group demonstrated significantly lower GLS LV than the closed group (−16.9 ± 3.7% vs. −15.1 ± 3.7%, *p* < 0.001) Table 2. After multivariable adjustment (ANCOVA, post adjusted for baseline TAPSE and prespecified covariates), pericardial closure was associated with higher postoperative TAPSE: AMD (Closed–Open) = 1.531 mm (95% CI 0.130–2.931; *p* = 0.033; Holm *p* = 0.033). For TASV, the adjusted effect was 1.694 cm/s (95% CI 0.437–2.951; *p* = 0.009; Holm p = 0.018) (Figure 1 and Table 3). Additionally, several right ventricular Doppler parameters were significantly different between the two groups, including early diastolic tricuspid inflow velocity (ETV), early diastolic tricuspid annular excursion velocity (E′), and late diastolic tricuspid annular excursion velocity (A′). The open pericardium group showed a significantly lower E′ (5.7 ± 2.9 cm/s vs. 6.7 ± 2.7 cm/s, *p* = 0.006) and A′ (5.7 ± 3.5 cm/s vs. 8.2 ± 3.3 cm/s, *p* < 0.001).

### 3.4. Correlation Between Open vs. Closed Pericardium and the Incidence of Postoperative Atrial Fibrillation

In our study cohort we could not detect a strong correlation between closing the pericardium and reducing the incidence of postoperative atrial fibrillation, we had a significant *p*-value of 0.047 but the Pearson’s r was only −0.116.

## 4. Discussion

In this retrospective cohort research of 97 patients undergoing isolated or combined coronary or/and aortic valve surgery, pericardial closure was associated with better preservation of early postoperative right-ventricular (RV) longitudinal function. Patients whose pericardium was closed had higher postoperative TAPSE (14.6 ± 3.1 mm vs. 12.9 ± 3.1 mm; *p* = 0.015) and higher TASV (9.8 ± 2.7 cm/s vs. 7.9 ± 2.5 cm/s; *p* = 0.003) than those whose pericardium was left open, and these differences persisted after adjustment for baseline characteristics. Postoperative LV GLS was also less depressed in the closed group (−15.1 ± 3.7% vs. −16.9 ± 3.7%; *p* < 0.001).

These results add to a heterogeneous body of evidence on the perioperative determinants of RV longitudinal indices. Several intraoperative TEE and speckle-tracking studies report an immediate decline in annular velocities coincident with pericardial opening, supporting the concept that loss of native pericardial restraint produces an abrupt geometric change that reduces longitudinal excursion [11,18,19].

At the same time, other prospective investigations using angle-independent speckle tracking and careful intraoperative sampling have found little or no change attributable solely to pericardial incision, suggesting that additional factors—cardiopulmonary bypass (CPB), myocardial ischemia, chest closure, or the surgical approach—substantially modulate the magnitude and timing of postoperative longitudinal declines [6,20,21].

In recent decades, significant discourse has emerged concerning right ventricular contraction patterns and their modifications under right ventricular stress [22]. In the context of cardiac surgery, numerous research studies have concentrated on the adaptive mechanisms related to right ventricular contraction following sternotomy, pericardiotomy, cardioplegia, and cardiopulmonary bypass [20,21]. Nevertheless, the evaluation of both established and novel metrics of right ventricular (RV) longitudinal function—such as tricuspid annular plane systolic excursion (TAPSE), longitudinal strain, or RV ejection fraction (RVEF) derived from longitudinal measurements—may obscure the reality that alternative measures, including three-dimensional (3D) derived RVEF or invasive hemodynamic data, could be more advantageous [23,24].

A significant study characterizing RV contraction patterns in patients undergoing elective CABG was recently reported by Donauer et al. [4]. The RV systolic function was maintained; however, RV longitudinal contraction, as assessed by 3D-derived strain analysis, was reduced, consistent with our findings.

Our finding that pericardial closure attenuated the fall in TAPSE and TASV is biologically plausible and consistent with historical and more recent reports that preserving pericardial integrity can influence RV geometry and hemodynamics after sternotomy [25,26]. Classic surgical series and technique papers have argued that pericardial closure prevents RV tethering to the sternum and may reduce postoperative adhesions and physiologic derangement after chest closure [27]. Conversely, multi-parameter studies have emphasized that decreases in longitudinal indices do not always reflect a true global loss of RV performance—global RV metrics (3D RVEF, RV-FAC, or invasive measures) can remain preserved despite marked TAPSE or S′ reductions—indicating a shift in contractile pattern rather than frank pump failure [7,19,26].

Taken together, these data suggest a multifactorial model in which pericardial disruption is an important but not exclusive driver of early longitudinal index reductions. The magnitude of change likely depends on the type and extent of pericardial incision/repair, myocardial protection strategy during CPB, chest-closure mechanics, and baseline ventricular geometry. Our results—showing preserved longitudinal RV-indices and less LV GLS depression with pericardial closure—support the hypothesis that maintaining pericardial continuity mitigates geometry-related loss of longitudinal shortening in the early postoperative window [26]. Furthermore, the concurrent preservation of LV GLS in the closed group suggests a beneficial interplay between the ventricles. One plausible mechanism is enhanced ventricular interdependence. By restoring the pericardial restraint, the geometry of the RV is better maintained, which in turn could prevent adverse diastolic shifting of the interventricular septum. This preserved septal geometry, and more coordinated biventricular motion likely contributes to more efficient LV mechanics and longitudinal shortening [28]. The pericardium provides an external constraint that stabilizes RV shape and septal position; when this restraint is preserved or restored, RV dilation and diastolic septal leftward shift (“D-shape”) are attenuated, which can improve LV filling geometry and subendocardial fiber shortening—plausibly explaining the less depressed LV GLS we observed in the closed group [29]. Classic and contemporary work on ventricular interdependence shows that septal position and pericardial restraint materially affect LV mechanics; even without overt RV failure, altered RV geometry can degrade LV systolic deformation via shared septal fibers and pericardial coupling. Pericardiotomy has been linked to an immediate fall in RV long-axis indices and paradoxical septal motion, whereas approaches that preserve or repair the pericardium mitigate these geometric disturbances—though not all studies agree, underscoring the multifactorial nature of perioperative changes (CPB, ischemia, chest closure). Collectively, our findings align with a model in which pericardial closure better maintains RV geometry and septal neutrality, thereby supporting LV deformation and yielding a smaller postoperative decrement in LV GLS [11,26].

### 4.1. Clinical Perspective

Pericardial closure after median sternotomy appears to be associated with better preservation of RV longitudinal function (TAPSE, TASV) and less LV GLS depression in the first postoperative week. Because longitudinal indices are sensitive to geometry and pericardial restraint, clinicians should avoid overinterpretation of isolated postoperative TAPSE or TASV declines and instead combine them with global RV measures (RV-FAC/3D RVEF/strain) and clinical endpoints when making management decisions. Our data support consideration of pericardial preservation or standardized closure techniques as part of protocols to protect RV performance after surgery; however, randomized data linking closure techniques to patient-centered outcomes are still lacking [11,30].

### 4.2. Limitations

This study has several important limitations that temper the interpretation of our findings. First, the retrospective, non-randomized design means that pericardial management was determined by surgeon`s preference rather than allocation, introducing potential selection bias and limiting causal inference. Second, baseline imbalances between groups—particularly in preoperative LV parameters—and the modest, uneven sample size reduce power for adjusted and subgroup analyses and increase the risk of residual confounding. Third, echocardiographic follow-up was restricted to the early postoperative period (within seven days), so we cannot address longer-term remodeling, functional recovery, or clinical outcomes such as heart-failure, rehospitalization, persistent RV failure, or mortality. Fourth, invasive hemodynamic data (e.g., Swan-Ganz measurements, standardized inotrope dosing) and systematic clinical endpoints were not available for all patients, which constrains our ability to determine whether the observed imaging differences translate into physiologically or clinically meaningful benefits. Fifth, the cohort was heterogeneous with respect to operative procedure (CABG, valve surgery, combined operations) and intraoperative management (cardioplegia strategy, CPB times, chest-closure mechanics), and these factors were not standardized or randomized; such heterogeneity may modify the relationship between pericardial handling and ventricular mechanics. Finally, although transthoracic speckle-tracking and tissue-Doppler analyses were used and reproducibility was assessed in a subset, early post-sternotomy imaging is technically challenging, and single-center acquisition/analysis may limit generalizability. Together, these limitations suggest caution in extrapolating our results and highlight the need for prospective, ideally randomized studies with standardized intraoperative protocols, multimodality imaging (including 3D echo/CMR), invasive hemodynamics, and longer follow-up to establish whether pericardial closure improves durable cardiac function or patient-centered outcomes.

## 5. Conclusions

In this retrospective cohort study of 97 patients, pericardial closure after median sternotomy was associated with better preservation of early postoperative RV longitudinal indices (TAPSE, TASV) and reduced depression of LV GLS compared with leaving the pericardium open. While these results are biologically plausible and suggest that pericardial management may be a modifiable factor to protect early ventricular mechanics, the observational design, baseline imbalances, and short follow-up prevent definitive causal claims. Prospective randomized trials and studies that combine 3D imaging, invasive hemodynamics, and patient-centered outcomes are warranted to determine whether pericardial closure improves durable cardiac function or clinical endpoints.

## Figures and Tables

**Table 1:** Showing comparison of echocardiographic changes between the two groups.

	Open (*n* = 32)	Closed (*n* = 65)	*p*-Values
**preoperative**			
LV GLS (%)	−19.2 ± 3.6	−17.4 ± 3.7	**<0.001**
LVEDD (mm)	47.7 ± 7.0	48.4 ± 8.3	0.479
LVEDVI (mL/m^2^)	48.6 ± 11.2	55.7 ± 24.3	**0.007**
LVEF (%)	58.1 ± 8.4	55.5 ± 8.9	**0.018**
SVI (mL/m^2^)	37.6 ± 12.4	37.1 ± 11.5	0.728
IVSd (mm)	13.6 ± 3.0	13.3 ± 3.1	0.683
RVEDD 1 (mm)	31.8 ± 7.0	33.0 ± 6.5	0.126
RVEDD 3 (mm)	80.5 ± 7.4	82.7 ± 10.1	0.052
TAPSE (mm)	21.6 ± 3.3	22.2 ± 4.4	0.558
TASV (cm/s)	11.7 ± 2.8	12.0 ± 3.6	0.709
Time to peak RV S′ (ms)	181.3 ± 43.7	187.6 ± 42.9	0.540
RVFAC (%)	41.5 ± 11.6	43.5 ± 10.0	0.382
Ratio TAPSE/RVFAC	0.6 ± 0.2	0.5 ± 0.2	0.506
RV Str	−1.5 ± 0.4	−1.6 ± 0.5	0.643
RV Strain (%)	−21.1 ± 6.0	−22.5 ± 5.8	0.320
PAPsys (mmHg)	19.9 ± 15.4	20.3 ± 11.0	0.880
RAP (mmHg)	18 ± 56.2	34 ± 52.3	0.480
VTI RVOT (cm)	16.5 ± 3.4	17.1 ± 3.7	0.582
Vmax TR (m/s)	1.9 ± 0.9	1.9 ± 0.6	0.502
PVR (wood units)	1.3 ± 0.9	1.3 ± 0.7	0.966
TAPSE/sPAP	1.0 ± 1.0	0.8 ± 0.6	0.233
E TV (cm/s)	60.8 ± 18.5	57.3 ± 11.7	0.074
A TV (cm/s)	50.6 ± 13.3	49.0 ± 10.9	0.334
E′ (cm/s)	8.4 ± 3.1	9.2 ± 4.0	0.091
A′ (cm/s)	11.7 ± 4.7	12.7 ± 5.6	0.184
E/A	1.2 ± 0.4	1.2 ± 0.3	0.672
E/E′	8.0 ± 3.8	6.9 ± 3.2	0.228
DeT TV (ms)	314.7 ± 116.2	339.7 ± 146.2	0.448
IVCT (ms)	74.5 ± 20.1	72.0 ± 19.4	0.741
IVRT (ms)	83.7 ± 30.3	83.3 ± 43.4	0.968
Ejection Time (ms)	278.1 ± 54.8	270.7 ± 49.3	0.530
Tei Index (TDI)	0.6 ± 0.3	0.6 ± 0.5	0.651
ET spectral RVOT (ms)	267.9 ± 58.6	369.8 ± 51.3	0.888
Transpulmonale T (ms)	295.0 ± 84.5	295.4 ± 81.3	0.982
Tei-Index (Spectral)	0.0 ± 0.6	0.0 ± 0.5	0.748
**Postoperative**			
GLS LV (%)	−15.1 ± 3.7	−16.9 ± 3.7	**<0.001**
LVEDD (mm)	48.7 ± 5.7	48.4 ± 7.5	0.697
LVEDVI (mL/m^2^)	47.4 ± 15.0	59.9 ± 69.8	0.093
LVEF (%)	53.3 ± 11.5	52.2 ± 9.3	0.435
SVI (mL/m^2^)	32.0 ± 10.6	31.1 ± 11.8	0.532
RVEDD 1 (mm)	33.5 ± 7.5	35.5 ± 6.4	0.170
RVEDD 3 (mm)	81.2 ± 9.3	82.0 ± 10.7	0.529
TAPSE (mm)	12.9 ± 3.1	14.6 ± 3.1	**0.015**
TASV (cm/s)	7.9 ± 2.5	9.8 ± 2.7	**0.003**
Time to peak RV S′ (ms)	147.1 ± 33.8	158.7 ± 31.8	0.187
RVFAC (%)	41.7 ± 15.8	42.2 ± 10.6	0.882
Ratio TAPSE/RVFAC	0.3 ± 0.2	0.4 ± 0.1	0.579
RV Str	−1.3 ± 0.8	−1.5 ± 1.5	0.311
RV Strain (%)	−14.2 ± 6.3	−15.3 ± 6.9	0.440
PAPsys (mmHg)	26.9 ± 21.4	23.4 ± 11.6	0.362
RAP (mmHg)	16 ± 50.0	30 ± 46.2	0.183
VTI RVOT (cm)	16.7 ± 5.5	14.5 ± 4.7	0.126
Vmax TR (m/s)	2.1 ± 0.9	2.0 ± 0.7	0.162
PVR (wood units)	1.2 ± 0.9	1.4 ± 0.9	0.443
TAPSE/sPAP	44.7 ± 42.2	39.1 ± 23.3	0.450
E TV (cm/s)	75.1 ± 20.0	63.3 ± 21.4	**<0.001**
A TV (cm/s)	52.6 ± 16.2	49.9 ± 18.3	0.282
E′ (cm/s)	5.7 ± 2.9	6.7 ± 2.7	**0.006**
A′ (cm/s)	5.7 ± 3.5	8.2 ± 3.3	**<0.001**
E/A	1.5 ± 0.4	1.3 ± 0.4	0.163
E/E′	18.4 ± 23.6	11.5 ± 9.2	0.089
DeT TV (ms)	303.5 ± 196.6	222.6 ± 80.6	**0.015**
IVCT (ms)	62.8 ± 15.0	66.0 ± 18.0	0.427
IVRT (ms)	78.1 ± 31.6	82.8 ± 34.6	0.545
Ejection Time (ms)	226.6 ± 65.4	205.6 ± 39.5	**0.070**
Tei Index (TDI)	0.7 ± 0.3	0.8 ± 0.3	**0.036**
ET spectral RVOT (ms)	248.0 ± 43.4	230.4 ± 41.2	0.145
Transpulmonale T (ms)	265.7 ± 61.8	253.3 ± 61.0	0.407
Tei Index Spektral	−0.1 ± 0.5	−0.1 ± 0.5	0.926

LV GLS, global longitudinal strain of the left ventricle; LVEDD, left ventricular end-diastolic diameter; LVEDVI, left ventricular end-diastolic volume index; LVEF, left ventricular ejection fraction; SVI, stroke volume index; RVEDD 1, right ventricular basal diameter at end-diastole; RVEDD 3, right ventricular longitudinal diameter at end-diastole; TAPSE, tricuspid annular plane systolic excursion; TASV, tricuspid annular systolic velocity; RVFAC, right ventricular fractional area change; RV Str, right ventricular strain rate; RV strain, right ventricular strain; PAPsys, pulmonary artery systolic pressure; RAP, right atrial pressure; VTI RVOT, right ventricular outflow tract velocity–time integral; Vmax TR, maximal tricuspid regurgitation velocity; PVR, pulmonary vascular resistance; E TV, early diastolic tricuspid inflow velocity; A TV, late diastolic tricuspid inflow velocity during atrial contraction; E′, early diastolic tricuspid annular velocity; A′, late diastolic tricuspid annular velocity during atrial contraction; DeT TV, E-wave deceleration time; IVCT, isovolumic contraction time; IVRT, isovolumic relaxation time; ET RVOT, ejection time over the right ventricular outflow tract; Transpulmonale T, transpulmonary time. Bold entries indicate significance.

**Table 2:** Comparison of echocardiographic changes within the groups.

	Open (*n* = 32)			Closed (*n* = 65)		
	Preoperative	Postoperative	*p* Values	Preoperative	Postoperative	*p* Values
LV GLS (%)	−19.246 ± 3.654	−16.879 ± 3.896	**0.006**	−17.589 ± 3.647	−15.023 ± 3.584	**<0.001**
LVEDD (mm)	46.773 ± 6.102	48.682 ± 5.875	1.000	48.263 ± 8.134	48.351 ± 7.499	1.000
LVEDVI (mL/m^2^)	48.910 ± 11.262	48.034 ± 14.949	1.000	56.878 ± 24.914	60.267 ± 70.746	1.000
LVEF (%)	58.379 ± 8.432	53.448 ± 11.776	0.164	55.492 ± 8.997	52.576 ± 9.058	0.283
SVI (mL/m^2^)	37.719 ± 12.775	32.032 ± 10.687	0.127	36.967 ± 11.463	31.323 ± 11.992	**0.019**
RVEDD 1 (mm)	31.750 ± 7.030	33.469 ± 7.577	0.707	33.319 ± 6.573	35.532 ± 6.467	0.175
RVEDD 3 (mm)	80.469 ± 7.457	81.156 ± 9.395	1.000	82.516 ± 10.300	81.968 ± 10.770	1.000
TAPSE (mm)	21.650 ± 3.300	12.869 ± 3.147	**<0.001**	22.197 ± 4.411	14.552 ± 3.103	**<0.001**
TASV (cm/s)	11.667 ± 2.817	8.329 ± 2.328	**<0.001**	12.009 ± 3.521	9.909 ± 2.708	**<0.001**
Time to peak RV S′ (ms)	168.059 ± 39.213	145.412 ± 33.577	0.072	185.310 ± 44.337	158.881 ± 33.207	**0.001**
RVFAC (%)	41.542 ± 11.634	41.750 ± 15.815	0.940	43.570 ± 9.885	41.159 ± 10.626	0.477
Ratio TAPSE/RVFAC	0.568 ± 0.211	0.347 ± 0.157	**<0.001**	0.541 ± 0.183	0.362 ± 0.113	**<0.001**
RV Str	−1.535 ± 0.383	−1.204 ± 0.849	0.267	−1.618 ± 0.512	−1.556 ± 1.508	0.745
RV Strain (%)	−21.074 ± 5.988	−13.552 ± 6.720	**<0.001**	−22.777 ± 5.755	−15.460 ± 6.779	**<0.001**
PAPsys (mmHg)	20.588 ± 16.567	28.832 ± 22.333	0.071	19.863 ± 10.628	22.802 ± 11.607	0.342
RAP (mmHg)	3.952 ± 2.012	5.333 ± 3.864	0.193	5.122 ± 3.120	6.122 ± 3.603	0.150
VTI RVOT (cm)	17.207 ± 3.761	16.450 ± 6.134	0.597	17.885 ± 3.596	14.565 ± 4.775	**<0.001**
Vmax TR (m/s)	1.866 ± 0.947	2.243 ± 0.955	0.567	1.891 ± 0.641	1.992 ± 0.720	1.000
PVR (wood units)	1.238 ± 0.612	1.387 ± 0.858	0.532	1.162 ± 0.448	1.361 ± 0.766	0.161
TAPSE/sPAP	1.044 ± 1.020	44.701 ± 42.223	**<0.001**	0.817 ± 0.633	39.060 ± 23.320	**<0.001**
E TV (cm/s)	50.455 ± 13.412	51.364 ± 16.552	1.000	48.690 ± 11.507	48.214 ± 13.377	1.000
A TV (cm/s)	50.455 ± 13.412	51.364 ± 16.552	0.807	48.690 ± 11.507	48.214 ± 16.377	0.860
E′ (cm/s)	8.535 ± 3.191	6.217 ± 2.621	**0.014**	9.362 ± 3.582	6.840 ± 2.788	**<0.001**
A′ (cm/s)	11.917 ± 5.147	6.304 ± 3.080	**<0.001**	13.055 ± 4.937	8.489 ± 3.053	**<0.001**
E/A	1.194 ± 0.288	1.513 ± 0.400	**0.006**	1.207 ± 0.268	1.327 ± 0.451	0.140
E/E′ (cm/s)	8.300 ± 4.007	12.218 ± 4.867	**0.048**	6.755 ± 3.126	11.478 ± 9.846	**<0.001**
DeT TV (ms)	318.636 ± 119.110	301.136 ± 181.893	0.630	354.214 ± 152.561	223.190 ± 84.594	**<0.001**
IVCT (ms)	72.458 ± 19.583	63.875 ± 12.585	0.099	72.321 ± 19.583	66.396 ± 18.137	0.091
IVRT (ms)	80.167 ± 27.886	79.625 ± 24.182	0.962	81.811 ± 45.349	83.849 ± 34.759	0.793
Ejection Time (ms)	276.542 ± 58.755	230.083 ± 61.679	**<0.001**	271.472 ± 47.013	205.585 ± 39.922	**<0.001**
Tei Index (TDI)	0.594 ± 0.271	0.686 ± 0.293	1.000	0.612 ± 0.487	0.776 ± 0.359	0.322
ET spectral RVOT (ms)	271.538 ± 67.002	242.615 ± 47.072	0.080	279.950 ± 45.034	232.025 ± 42.225	**<0.001**
Transpulmonale T (ms)	290.045 ± 88.245	263.591 ± 60.970	0.207	288.957 ± 88.241	251.957 ± 60.582	**0.012**
Tei Index (Spectral)	0.081 ± 0.499	0.027 ± 0.410	1.000	0.046 ± 0.356	−0.101 ± 0.470	0.818

LV GLS, Global longitudinal strain left ventricle; LVEDD, Left ventricular end-diastolic diameter; LVEDVI, Left ventricular end-diastolic volume index; LVEF, left ventricular ejection fraction; SVI, Stroke volume index; RVEDD 1, right ventricular basal diameter at end-diastole; RVEDD 3, right ventricular longitudinal diameter at end-diastole; TAPSE, tricuspid annular plane systolic excursion; TASV, tricuspid annular systolic velocity; RVFAC, right ventricular fractional area change; RV Str, right ventricular Strain rate; RV Strain, right ventricular strain; PAPsys, pulmonary artery systolic pressure; RAP, right atrial pressure; VTI RVOT, Right ventricular outflow tract velocity time integral; Vmax TR, maximal tricuspid regurgitation velocity; PVR, pulmonary vascular resistance; E TV, early diastolic tricuspid inflow velocity; A TV, late diastolic tricuspid inflow velocity during atrial contraction; E′, early diastolic tricuspid annular excursion velocity; A′, late diastolic tricuspid annular excursion velocity during atrial contraction; DeT TV, E-Wave deceleration time; IVCT, isovolumetric contraction time; IVRT, isovolumetric relaxation time; ET spectral RVOT, ejection time over right ventricular outflow tract; Transpulmonale T, transpulmonary time. Bold entries indicate significance.

**Table 3:** Adjusted Effects of Pericardial Closure on Early RV Longitudinal Function.

Outcome	AMD (Closed–Open)	95% CI	*p*	*p* (Holm)
TAPSE (mm)	1.531	0.130 to 2.931	0.033	0.033
TASV (cm/s)	1.694	0.437 to 2.951	0.009	0.018

Abbreviations: AMD, adjusted mean difference; CI, confidence interval; RV, right ventricle; TAPSE, tricuspid annular plane systolic excursion; TASV, tricuspid annular systolic velocity. Models were analysis-of-covariance (ANCOVA) with group (Closed vs. Open) as exposure, adjusted for baseline value of the outcome and prespecified covariates (LV GLS, LVEF, LVEDVI, sex, procedure; CPB and cross-clamp time when available). Holm correction was applied across the two co-primary endpoints (TAPSE and TASV).

## Data Availability

The data that support the findings of this study are available on request from the corresponding author. The data are not publicly available due to privacy or ethical restrictions.

## Abbreviations

The following abbreviations are used in this manuscript:

(2D-)STE	(Two-dimensional) Speckle-tracking Echocardiography
3D	Three-dimensional
4AC	Apical Four Chamber View
5AC	Apical Five Chamber View
A (TV)	Late Diastolic Tricuspid Inflow Velocity during Atrial Contraction
A′	Late Diastolic Tricuspid Annular velocity during Atrial Contraction
AF	Atrial Fibrillation
APLAX	Apical Long Axis
AUC	Area Under the Curve
AVR	Aortic Valve Replacement
CABG	Coronary Artery Bypass Grafting
CMR	Cardiovascular Magnetic Resonance
COPD	Chronic Obstructive Pulmonary Disease
CPB	Cardiopulmonary Bypass
DeT TV	E-wave Deceleration Time
E (TV)	Early Diastolic Tricuspid Inflow Velocity
E′	Early Diastolic Tricuspid Annular Velocity
ECG	Electrocardiography
ET	Ejection Time
ET RVOT	Ejection Time over the Right Ventricular Outflow Tract
IVCT	Isovolumetric Contraction Time
IVRT	Isovolumetric Relaxation Time
LV	Left Ventricle
LV GLS	Left Ventricular Global Longitudinal Strain
LVEDD	Left Ventricular End-Diastolic Diameter
LVEDVI	Left Ventricular End-Diastolic Volume Index
LVEF	Left-Ventricular Ejection Fraction
LVOTd	Left Ventricular Outflow Tract Diameter
LVSVI	Left Ventricular Stroke Volume Index
MPI	Myocardial Performance Index
PAPsys	Pulmonary Artery Systolic Pressure
PLAX	Parasternal Long Axis
PSAX	Parasternal Short Axis
PVR	Pulmonary Vascular Resistance
PW-TDI	PW-Tissue Doppler Imaging
RA	Right Atrium
RAP	Right Atrial Pressure
ROC	Receiver Operating Characteristic
RV	Right Ventricle
RV ET	Right Ventricular Ejection Time
RV Str	Right Ventricular StrainRate
RV Strain	Right Ventricular Strain
RVEDD1	Right Ventricular Basal Diameter at End-Diastole
RVEDD3	Right Ventricular Longitudinal diameter at End-Diastole
RVEF	Right Ventricular Ejection Fraction
RVF	Right Ventricular Function
RV-FAC	Right Ventricular Fractional Area Change
SD	Standard Deviation
SVI	Stroke Volume Index
TAPSE	Tricuspid Annular Plane Systolic Excursion
TASV, S′	Tricuspid Annular Systolic Velocity
TEE	Transesophageal echocardiography
Transpulmonale T	Transpulmonary Time
TRVmax	Maximum Flow Velocity of Systolic Tricuspid Regurgitation
TTE	Transthoracic Echocardiography
VCI	Vena Cava Inferior
VTI	Velocity-Time Integral

## References

[1] Purmah Y., Lei L.Y., Dykstra S., Mikami Y., Cornhill A., Satriano A., Flewitt J., Rivest S., Sandonato R., Seib M. (2021). Right Ventricular Ejection Fraction for the Prediction of Major Adverse Cardiovascular and Heart Failure-Related Events. Circ. Cardiovasc. Imaging.

[2] Denault A., Haddad F., Lamarche Y., Bouabdallaoui N., Deschamps A., Desjardins G. (2020). Postoperative right ventricular dysfunction-Integrating right heart profiles beyond long-axis function. J. Thorac. Cardiovasc. Surg..

[3] Towheed A., Sabbagh E., Gupta R., Assiri S., Chowdhury M.A., Moukarbel G.V., Khuder S.A., Schwann T.A., Bonnell M.R., Cooper C.J. (2021). Right Ventricular Dysfunction and Short-Term Outcomes Following Left-Sided Valvular Surgery: An Echocardiographic Study. J. Am. Heart Assoc..

[4] Donauer M., Schneider J., Jander N., Beyersdorf F., Keyl C. (2020). Perioperative Changes of Right Ventricular Function in Cardiac Surgical Patients Assessed by Myocardial Deformation Analysis and 3-Dimensional Echocardiography. J. Cardiothorac. Vasc. Anesth..

[5] Haddad A., Mohamed A., Arends S., Ishak B., Tsarenko O., Balaj I., Kamler M., Brenner T., Shehada S.E. (2024). Echocardiographic right ventricular evaluation in cardiac surgery patients undergoing mitral valve reconstruction: A single center prospective observational study. J. Thorac. Dis..

[6] Bitcon C.J., Tousignant C. (2017). The effect of pericardial incision on right ventricular systolic function: A prospective observational study. Can. J. Anaesth..

[7] Bootsma I.T., Scheeren T.W.L., de Lange F., Jainandunsing J.S., Boerma E.C. (2020). The Reduction in Right Ventricular Longitudinal Contraction Parameters Is Not Accompanied by a Reduction in General Right Ventricular Performance During Aortic Valve Replacement: An Explorative Study. J. Cardiothorac. Vasc. Anesth..

[8] Gaudino M., Pragliola C., Anselmi A., Pieroni M., De Paulis S., Leone A., De Caterina A.R., Massetti M. (2013). Randomized trial of HTK versus warm blood cardioplegia for right ventricular protection in mitral surgery. Scand. Cardiovasc. J..

[9] Merlo A., Cirelli C., Vizzardi E., Fiorendi L., Roncali F., Marino M., Merlo M., Senni M., Sciatti E. (2024). Right Ventricular Dysfunction before and after Cardiac Surgery: Prognostic Implications. J. Clin. Med..

[10] Unsworth B., Casula R.P., Yadav H., Baruah R., Hughes A.D., Mayet J., Francis D.P. (2013). Contrasting effect of different cardiothoracic operations on echocardiographic right ventricular long axis velocities, and implications for interpretation of post-operative values. Int. J. Cardiol..

[11] Unsworth B., Casula R.P., Kyriacou A.A., Yadav H., Chukwuemeka A., Cherian A., de Lisle Stanbridge R., Athanasiou T., Mayet J., Francis D.P. (2010). The right ventricular annular velocity reduction caused by coronary artery bypass graft surgery occurs at the moment of pericardial incision. Am. Heart J..

[12] Mauermann E., Vandenheuvel M., François K., Bouchez S., Wouters P. (2020). Right Ventricular Systolic Assessment by Transesophageal Versus Transthoracic Echocardiography: Displacement, Velocity, and Myocardial Deformation. J. Cardiothorac. Vasc. Anesth..

[13] Geyer H., Caracciolo G., Abe H., Wilansky S., Carerj S., Gentile F., Nesser H.J., Khandheria B., Narula J., Sengupta P.P. (2010). Assessment of myocardial mechanics using speckle tracking echocardiography: Fundamentals and clinical applications. J. Am. Soc. Echocardiogr..

[14] Voigt J.U., Pedrizzetti G., Lysyansky P., Marwick T.H., Houle H., Baumann R., Pedri S., Ito Y., Abe Y., Metz S. (2015). Definitions for a common standard for 2D speckle tracking echocardiography: Consensus document of the EACVI/ASE/Industry Task Force to standardize deformation imaging. Eur. Heart J. Cardiovasc. Imaging.

[15] Lang R.M., Badano L.P., Mor-Avi V., Afilalo J., Armstrong A., Ernande L., Flachskampf F.A., Foster E., Goldstein S.A., Kuznetsova T. (2016). Recommendations for Cardiac Chamber Quantification by Echocardiography in Adults: An Update from the American Society of Echocardiography and the European Association of, Cardiovascular Imaging. Eur. Heart J. Cardiovasc. Imaging.

[16] Negishi K., Negishi T., Kurosawa K., Hristova K., Popescu B.A., Vinereanu D., Yuda S., Marwick T.H. (2015). Practical guidance in echocardiographic assessment of global longitudinal strain. JACC Cardiovasc. Imaging.

[17] Rudski L.G., Lai W.W., Afilalo J., Hua L., Handschumacher M.D., Chandrasekaran K., Solomon S.D., Louie E.K., Schiller N.B. (2010). Guidelines for the echocardiographic assessment of the right heart in adults: A report from the American Society of Echocardiography endorsed by the European Association of Echocardiography, a registered branch of the European Society of Cardiology, and the Canadian Society of Echocardiography. J. Am. Soc. Echocardiogr..

[18] Raina A., Vaidya A., Gertz Z.M., Susan C., Forfia P.R. (2013). Marked changes in right ventricular contractile pattern after cardiothoracic surgery: Implications for post-surgical assessment of right ventricular function. J. Heart Lung Transplant..

[19] Tamborini G., Muratori M., Brusoni D., Celeste F., Maffessanti F., Caiani E.G., Alamanni F., Pepi M. (2009). Is right ventricular systolic function reduced after cardiac surgery? A two- and three-dimensional echocardiographic study. Eur. J. Echocardiogr..

[20] Korshin A., Grønlykke L., Nilsson J.C., Møller-Sørensen H., Ihlemann N., Kjøller S.M., Damgaard S., Lehnert P., Hassager C., Kjaergaard J. (2019). Tricuspid annular plane systolic excursion is significantly reduced during uncomplicated coronary artery bypass surgery: A prospective observational study. J. Thorac. Cardiovasc. Surg..

[21] Singh A., Huang X., Dai L., Wyler D., Alfirevic A., Blackstone E.H., Pettersson G.B., Duncan A.E. (2020). Right ventricular function is reduced during cardiac surgery independent of procedural characteristics, reoperative status, or pericardiotomy. J. Thorac. Cardiovasc. Surg..

[22] Kovács A., Lakatos B., Tokodi M., Merkely B. (2019). Right ventricular mechanical pattern in health and disease: Beyond longitudinal shortening. Heart Fail. Rev..

[23] Vitarelli A., Mangieri E., Terzano C., Gaudio C., Salsano F., Rosato E., Capotosto L., D’Orazio S., Azzano A., Truscelli G. (2015). Three-Dimensional Echocardiography and 2D-3D Speckle-Tracking Imaging in Chronic Pulmonary Hypertension: Diagnostic Accuracy in Detecting Hemodynamic Signs of Right Ventricular (RV) Failure. J. Am. Heart Assoc..

[24] Lee J.Z., Low S.W., Pasha A.K., Howe C.L., Lee K.S., Suryanarayana P.G. (2018). Comparison of tricuspid annular plane systolic excursion with fractional area change for the evaluation of right ventricular systolic function: A meta-analysis. Open Heart.

[25] Keller M., Heller T., Lang T., Patzelt J., Schreieck J., Schlensak C., Rosenberger P., Magunia H. (2020). Acute changes of global and longitudinal right ventricular function: An exploratory analysis in patients undergoing open-chest mitral valve surgery, percutaneous mitral valve repair and off-pump coronary artery bypass grafting. Cardiovasc. Ultrasound.

[26] Zanobini M., Loardi C., Poggio P., Tamborini G., Veglia F., Di Minno A., Myasoedova V., Mammana L.F., Biondi R., Pepi M. (2018). The impact of pericardial approach and myocardial protection onto postoperative right ventricle function reduction. J. Cardiothorac. Surg..

[27] Rao V., Komeda M., Weisel R.D., Cohen G., Borger M.A., David T.E. (1999). Should the pericardium be closed routinely after heart operations?. Ann. Thorac. Surg..

[28] Santamore W.P., Dell’Italia L.J. (1998). Ventricular interdependence: Significant left ventricular contributions to right ventricular systolic function. Prog. Cardiovasc. Dis..

[29] Borlaug B.A., Reddy Y.N. (2019). The role of the pericardium in heart failure: Implications for pathophysiology and treatment. JACC Heart Fail..

[30] David J.S., Tousignant C.P., Bowry R. (2006). Tricuspid annular velocity in patients undergoing cardiac operation using transesophageal echocardiography. J. Am. Soc. Echocardiogr..

